# Link between Morphology, Structure, and Interactions of Composite Microgels

**DOI:** 10.1021/acs.macromol.1c02171

**Published:** 2022-02-14

**Authors:** Rodrigo Rivas-Barbosa, José Ruiz-Franco, Mayra A. Lara-Peña, Jacopo Cardellini, Angel Licea-Claverie, Fabrizio Camerin, Emanuela Zaccarelli, Marco Laurati

**Affiliations:** † Department of Physics, Sapienza University of Rome, Piazzale Aldo Moro 2, 00185 Roma, Italy; ‡ División de Ciencias e Ingenierías, Universidad de Guanajuato, Lomas del Bosque 103, 37150 León, Mexico; § CNR Institute of Complex Systems, Uos Sapienza, Piazzale Aldo Moro 2, 00185 Roma, Italy; ∥ Physical Chemistry and Soft Matter, Wageningen University & Research, Stippeneng 4, 6708WE Wageningen, The Netherlands; ⊥ Dipartimento di Chimica and CSGI, Universitá di Firenze, 50019 Sesto Fiorentino, Italy; ▲ Centro de Graduados e Investigación en Química del Tecnológico Nacional de México, Instituto Tecnológico de Tijuana, 22500 Tijuana, Mexico

## Abstract

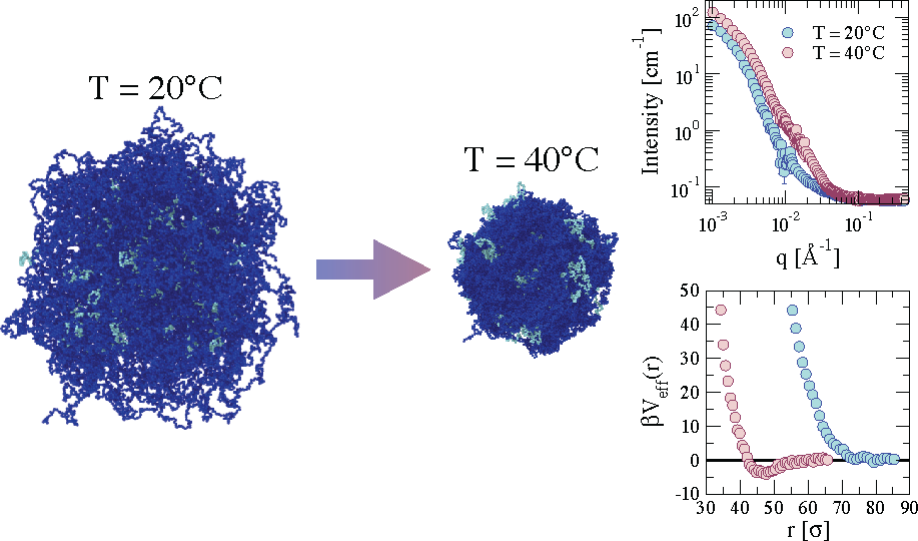

We combine small-angle scattering experiments and simulations to investigate the internal structure and interactions of composite poly(*N*-isopropylacrylamide)–poly(ethylene glycol) (PNIPAM–PEG) microgels. At low temperatures the experimentally determined form factors and the simulated density profiles indicate a loose internal particle structure with an extended corona that can be modeled as a starlike object. With increasing temperature across the volumetric phase transition, the form factor develops an inflection that, using simulations, is interpreted as arising from a conformation in which PEG chains are incorporated in the interior of the PNIPAM network. This gives rise to a peculiar density profile characterized by two dense, separated regions, at odds with configurations in which the PEG chains reside on the surface of the PNIPAM core. The conformation of the PEG chains also have profound effects on the interparticle interactions: Although chains on the surface reduce the solvophobic attraction typically experienced by PNIPAM particles at high temperatures, PEG chains inside the PNIPAM network shift the onset of attractive interaction at even lower temperatures. Our results show that by tuning the morphology of the composite microgels, we can qualitatively change both their structure and their mutual interactions, opening the way to explore new collective behaviors of these objects.

## Introduction

Soft polymeric colloids display properties that are determined by the interplay between colloidal behavior and the features of the internal polymeric structure.^1^ The internal structure not only affects the single-particle properties but also influences the particle–particle interactions.^2^ Within the family of polymeric soft colloids, microgels, in which the internal structure is made of a cross-linked polymer network with a typical core–corona architecture,^3^ is a widely investigated system. The polymer–colloid duality of this model system can be exploited to tackle fundamental physics problems, such as glass and jamming transition,^4−7^ as well as to develop wide-ranging applications, including drug delivery systems,^8^ inks for 3D printing,^9^ systems for CO_2_ capture,^10^ and regenerative scaffolds.^11^

The properties of microgels strongly depend on the nature of the constituent polymers, which determines how the microgels respond to the variation of, for instance, temperature,^3^ pH,^12^ or external fields.^13^ Most studies have focused on thermoresponsive microgels made of poly(*N*-isopropylacrylamide) (PNIPAM),^14,15^ whose hallmark is the presence of the so-called volume phase transition (VPT) in water at a characteristic temperature *T*_c_ ∼ 32 °C from a swollen state at low *T* to a compact state at high *T*. This transition is linked to changes in the mechanical properties of the particles:^16^ whereas the colloid is soft in the swollen state, it becomes stiffer above *T*_c_, where also the presence of attractive interactions arises, leading ultimately to phase separation.^17^ This description can be modified by adding ionic groups,^13,18^ inducing nonspherical shapes during the synthesis process,^19−21^ or creating core/shell microgels.^22−24^ Thus, microgels not only display a self-adaptive behavior to environmental changes but can also be programmed to have a specific response thanks to the precise knowledge of the topology of the network and to the different polymers used during the synthesis. In this way, the spectrum of microgel applications can even be enlarged, covering photonic devices,^25^ regenerative materials,^26^ and biomaterial design,^27^ to name a few.

Among the wide range of possible modifications, the inclusion of poly(ethylene glycol) (PEG) in the PNIPAM microgel network has the potential of increasing the biocompatibility of the particles for drug delivery applications, and can also be used to tune the value of *T*_c_ and the degree of deswelling associated with the VPT.^28,29^ However, the microscopic origin of these phenomena is yet unclear. Because PEG can be considered unaffected by *T* in the range where the VPT occurs, these effects must be related to the relative distributions of PEG and PNIPAM within the particles, to their interactions and to how these affect the internal structure as a function of *T*.

To shed light on these mechanisms, in this work we investigate composite microgels of PNIPAM and PEG using a combination of experiments and numerical simulations. We characterize the effect of PEG chains on the morphology of the microgels across the VPT using small-angle neutron (SANS) and X-ray (SAXS) scattering experiments. Numerical simulations are then used to rationalize the experimental findings by studying how the distribution and conformations of PEG chains in the particles affect the PNIPAM network structure as a function of *T*. In particular, we show that although the presence of PEG inside the composite microgel induces formation of two dense regions and a smaller particle size, the size of the particle increases when the PEG chains are distributed on the surface. A qualitative comparison with experimental results allows us to discriminate that, for the composite microgels investigated in this study, the PEG chains are mostly located inside the PNIPAM network. We also calculate the effective potential for each distribution, finding different behaviors depending on the PEG chains arrangement. On one hand, we find that the addition of PEG chains on the surface of the microgels induces repulsive interactions, even at temperatures above the VPT, thus effectively shielding the hydrophobic attraction between PNIPAM monomers. On the other hand, when the PEG chains are inside the microgels, attractive interactions arise, even below the VPT, at odds with standard PNIPAM microgels. These results suggest that tuning the microgel morphology is a convenient way to tailor the structure and the interactions between the particles, which can be exploited in the future to vary the assembly and the rheology of these systems at high densities.

## Materials and Methods

### In Vitro PNIPAM–PEG Particles

#### Microgel Synthesis

Composite PNIPAM–PEG microgel particles were synthesized following a “one-pot” soapless emulsion polymerization method.^28^ All reagents were purchased from Sigma-Aldrich. *N*-Isopropylacrylamide (*M*_n_ = 113.16 g/mol) was purified by recrystallization in petroleum ether at 35 °C. The cross-linker ethylene glycol dimethacrylate (EGDMA), the initiator ammonium persulfate (APS) (*M*_n_ = 228.18 g/mol), and the poly(ethylene glycol) methyl ether methacrylate (PEG) (*M*_n_ = 950 g/mol) were used as purchased. The synthesis was carried out using a 1 L jacketed glass reactor (Syrris, model Atlas Potassium, Royston, U.K.) to improve the temperature and stirring control. The particles were synthesized with a proportion in weight equal to 30% PEG and 70% PNIPAM. Initially, 3.5 g of PNIPAM was dissolved in 40 mL of water and mixed with the EGDMA cross-linker (1 mol % vs PNIPAM). The so-obtained solution was bubbled with nitrogen for 30 min to remove any dissolved oxygen while being stirred at 350 rpm in a cold bath at 15 °C. After 20 min, 1.5 g of PEG predissolved in 10 mL of water was added to the solution, and the bubbling was maintained for 10 additional minutes. The obtained mixture was added to 438 mL of preheated water (85 °C) and stirred at 350 rpm for 30 min. APS (2 wt % vs PNIPAM) previously dissolved in 12 mL of water was added to initiate the reaction. The polymerization was carried for 45 min, after which the solution was placed in a cold bath to stop the polymerization process. The dispersion was purified via dialysis for 7 days, and the microgel particles were recovered by freeze-drying. The microgel particles were redispersed in deuterated water (D_2_ O), resulting in a diluted sample with concentration *C* = 0.0010 g/mL. Deuterated water was chosen to increase the contrast in neutron scattering measurements. Particle characterization obtained by dynamic light scattering in a previous work showed that the hydrodynamic radius *R*_H_ ≈ 166 nm at small *T* and that the volume phase transition (VPT) occurs at *T*_c_ ≈ 36 °C, leading to *R*_H_ ≈ 90 nm at high *T*.^30^ This value of *T*_c_ is sensibly larger than that usually found for standard PNIPAM microgels (*T*_c_ ≈ 32 °C).

#### Small-Angle Neutron Scattering (SANS) Measurements

SANS measurements were performed at the NG7 SANS beamline (NCNR at NIST, Gaithersburg, MD, USA) using three different configurations: (i) 1.33 m sample-to-detector distance (SDD) and incident wavelength λ = 6 Å, (ii) 4 m SDD and λ = 6 Å, and (iii) 13.17 m SDD and λ = 8.4 Å. The combination of the three configurations gives a wave vector range 0.001 < *q* < 0.4 Å^–1^. The neutrons were detected with ^3^He 640 × 640 mm position-sensitive counters with a 5.08 × 5.08 mm resolution. The beam wavelength spread is Δλ/λ = 0.138. The scattering length density of the different components of the samples were determined using the NIST scattering length density calculator (https://www.ncnr.nist.gov/resources/activation/): ρ_PNIPAAm_ = 0.814 × 10^–6^ Å^–2^, ρ_PEG_ = 0.599 × 10^–6^ Å^–2^, and ρ_D_2_O_ = 6.38 × 10^–6^ Å^–2^. Measurements were performed at 20, 30, and 40 °C.

#### Small-Angle X-ray Scattering (SAXS) Measurements

The SAXS experiments were performed at the Austrian SAXS Beamline at Elettra Sincrotrone Trieste (Trieste, Italy). X-ray photons of energy 8 keV, corresponding to a wavelength λ = 0.154 nm, were used in the experiments. The *q* range of the measurements was 0.035 < *q* < 0.8 Å^–1^. The sample was measured at 25, 30, 35, 40, 45, 50, 55, and 60 °C. Intensities from samples were corrected for the empty cell and solvent contributions.

#### SANS Data Analysis

The intensity profile or macroscopic cross section in a neutron scattering experiment on dispersions of colloidal particles is given by^31^
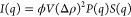
where ϕ is the volume fraction occupied by the particles, *V* the particle volume, Δρ = ρ_1_ – ρ_2_ the scattering length density difference between the particles (ρ_1_) and the solvent (ρ_2_), *P*(*q*) the particle form factor, and *S*(*q*) the structure factor. For dilute samples, as in this work, *S*(*q*) = 1 and the scattered intensity is proportional to the particle form factor *P*(*q*). Considering the small degree of cross-linking of the PNIPAM component, we expect a very open particle structure. For this reason, following previous work on PNIPAM microgel particles cross-linked with PEG^32^ having a similar cross-linker density, we used the star polymer form factor model of Dozier and co-workers^33^ to fit the experimental intensity profiles. The model consists of two terms:
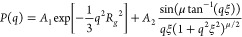
1The first term is a Guinier form factor, which yields a measure of the size of the particles through the radius of gyration *R*_g_. Polydispersity in the *R*_g_ value was included by considering a Gaussian distribution of this quantity with a width σ that was determined from fitting. The second term models the blob scattering of the star arms. The excluded volume correlation length or blob size ξ is the characteristic length scale at which the granular polymer structure becomes relevant. The quantity μ is defined as μ = 1/ν – 1, where ν is the Flory exponent. The amplitudes *A*_1_ and *A*_2_ weigh the contributions of the total and internal terms of the model. Smearing contributions were included in the fitting procedure through convolution of the form factor with a smearing function:
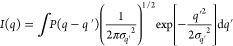
2where σ_q_ is the standard deviation of the *q* resolution, which encloses both the detector resolution and the beam wavelength spread contributions.^34^ Data modeling was performed with SasView.^35^

### In Silico PNIPAM–PEG Particles

#### Numerical Microgel Synthesis

Previous well-established protocols were followed:^36^ microgels were numerically designed as fully bonded, disordered networks resulting from the self-assembly of a binary mixture of limited-valence particles of diameter σ_m_. Specifically, we used *N*_A_ particles of species A with two attractive patches to mimic monomers (*N*-isopropylacrylamide, NIPAM) and *N*_B_ particles of species B with four attractive patches to resemble cross-linkers (ethylene glycol dimethacrylate, EGDMA). To reproduce the characteristic core–corona structure of the microgels, we also used an additional confining force acting on the cross-linkers only.^37^ Once a fully bonded network was obtained, the topology of the structure was fixed by making bonds permanent. To do this, the patchy interactions were replaced by ones representative of polymeric systems, by using the Kremer-Grest bead–spring model,^38^ where all particles interact via a Weeks–Chandler–Andersen (WCA) potential, defined as
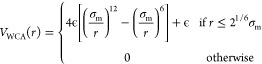
3where σ_m_ is the unit of length and ϵ controls the energy scale. Additionally, bonded particles also interact via a FENE potential, *V*_FENE_:
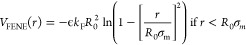
4where *k*_F_ = 15 is the dimensionless spring constant and *R*_0_ = 1.5 is the maximum extension of the bond.

Finally, the thermoresponsive behavior of the PNIPAM microgels is captured by adding an effective attraction among monomers:
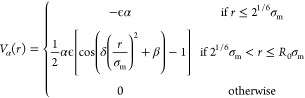
5with δ = π(2.25 – 2^1/3^)^−1^ and β = 2π – 2.25δ.^39^ The parameter α modulates the solvophobicity of the beads and plays the role of an effective temperature in the simulations:^39,40^ for α = 0, the effective attraction is not present, and hence we can reproduce good solvent conditions. Previous works have shown that the VPT transition occurs at a critical value, α_c_ ∼ 0.65.^36,41^

#### Addition of PEG Chains

Once the microgel is formed, we perform a second step in the numerical synthesis to incorporate the PEG chains into the polymeric network. To compare with the experimental observations, we consider three possible ways of distributing the chains within the swollen microgel, that is, at α = 0: (i) one end of each chain is attached to a NIPAM monomer on the surface of the microgel, whereas the other end remains free; (ii) both ends of each chain are attached to PNIPAM monomers on the surface of the microgel; (iii) chains are inserted within the microgel, allowing them to find accommodation for all beads via energy minimization. The system is then relaxed, and we allow both ends of the chains to form links with PNIPAM monomers in the network. In this work, we refer to these three distributions conventionally as *chains*, *loops*, and *inside*, respectively.

The interaction between PNIPAM and PEG also follows the Kremer–Grest bead spring model; however, for the PEG monomers, the effective attraction due to the thermoresponsivity is ignored because it is well-known that solvent quality effects become evident at a much higher temperature than that for PNIPAM ones,^42^ outside the effective temperature range investigated in this work.

#### Determination of Structural Quantities

The microgel radius of gyration is defined as
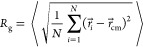
6where the brackets ⟨ ⟩ indicate ensemble averages, *r⃗*_*i*_ is the position of the *i*th monomer, and *r⃗*_cm_ is the microgel’s center of mass.

The inner structure of the macromolecules was studied through the radial density profile:
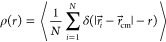
7At the same time, the microgel form factor *P*(*q*) was calculated from equilibrated trajectories using the following expression:
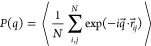
8where *r⃗*_*ij*_ is the distance between monomers *i* and *j*. Here, angular brackets indicate an average over different configurations and orientations of the wave vector *q⃗*. In particular, we consider 300 distinct directions randomly chosen on a sphere of radius *q*.

#### Simulation Parameters

To match the experimental polydispersity, we simulated microgels with *N* = 5000, 20000, 31000, and 42000 beads, all of them at a cross-linker concentration *c* = 1%. Then to fix the number of PEG chains *f* and their contour length, defined as *L*_c_ = *N*_pol_*b*, we run a set of simulations at α = 0 for the case where the chains are attached to the microgel by one end. Here, *N*_pol_ corresponds to the number of beads and *b* is the minimum of the FENE interaction. In particular, we fixed *f* and *N*_pol_^chains^ so that ρ(*r*) → 0 happens at the same *L*_c_^chains^/*R*_g_^M^ for the four different microgels considered here, where *R*_g_^M^ refers to the radius of gyration for the microgel without PEG chains. On the other hand, for the loops case, we considered *L*_c_^loops^ = 2*L*_c_^chains^ to ensure that ρ(*r*) decays approximately at the same *r* value as that for the chains case. Finally, for the inside distribution, PEG chains were cut to guarantee that *L*_c_^inside^ < *R*_g_^M^. The simulation parameters employed in this work were chosen to make the modeling similar to experiments but also feasible and are reported in Table 1. For the chains case, the number of PEG monomers in the simulations is approximately 25% of the total for a *N* = 42000 microgel, very close to the experimental value. In addition, we note that the internal structure of the microgel (for the chains case) was not affected by varying *f* in the range *f* ∈ [90, 240]. Hence, we decided to set this parameter to the minimum value (*f* = 90), which allows us to qualitatively explore the influence of PEG on the polymeric network of PNIPAM for the three distributions considered here.

**Table 1:** Summary of Simulation Model Parameters

*N*_microgel_	*f*	*N*_pol_^chains^	*N*_pol_^loops^	*N*_pol_^inside^
5000	90	10	20	4
20000	90	70	140	28
31000	90	90	180	36
42000	90	120	240	48

We perform molecular dynamics (MD) simulations of each composite microgel at different α values by using a Langevin thermostat to set the reduced temperature T* = *k*_B_*T*/ϵ = 1. All beads have unit mass *m*, and the integration time step is . Once the simulations are properly equilibrated for each α, we measure the observables explained above, and then we average them over microgels with different sizes to reproduce, at best, a similar polydispersity to that of the experimental sample. All simulations are made with LAMMPS.^43^

#### Assessment of the Effective Interaction Potential

The two-body effective potential between two composite microgels is evaluated by means of the umbrella sampling technique, where a series of independent configurations along a reaction coordinate are sampled by using a bias potential.^44,45^ In this work, we consider the centers of mass distance of the macromolecules as the reaction coordinate and the bias potential to be harmonic. Then we evaluate the bias probability distribution *P*_b_(*r*,Δ_*i*_) of finding the macromolecules’ centers of mass at distance *r* given the equilibrium length of the spring μ_*i*_ from our simulations. Later, the contribution from the bias potential is removed, *P*_u_(*r*,Δ_*i*_), and subsequently unbiased probability distributions are merged into *P*(*r*) via a least-squares method. Thus, the potential of the mean force is expressed as
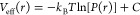
9with *C* being a constant that is set by imposing the condition *V*_eff_(*r*→∞) = 0.

## Results

### Experimental Form Factor Obtained from SANS

**Figure 1:**
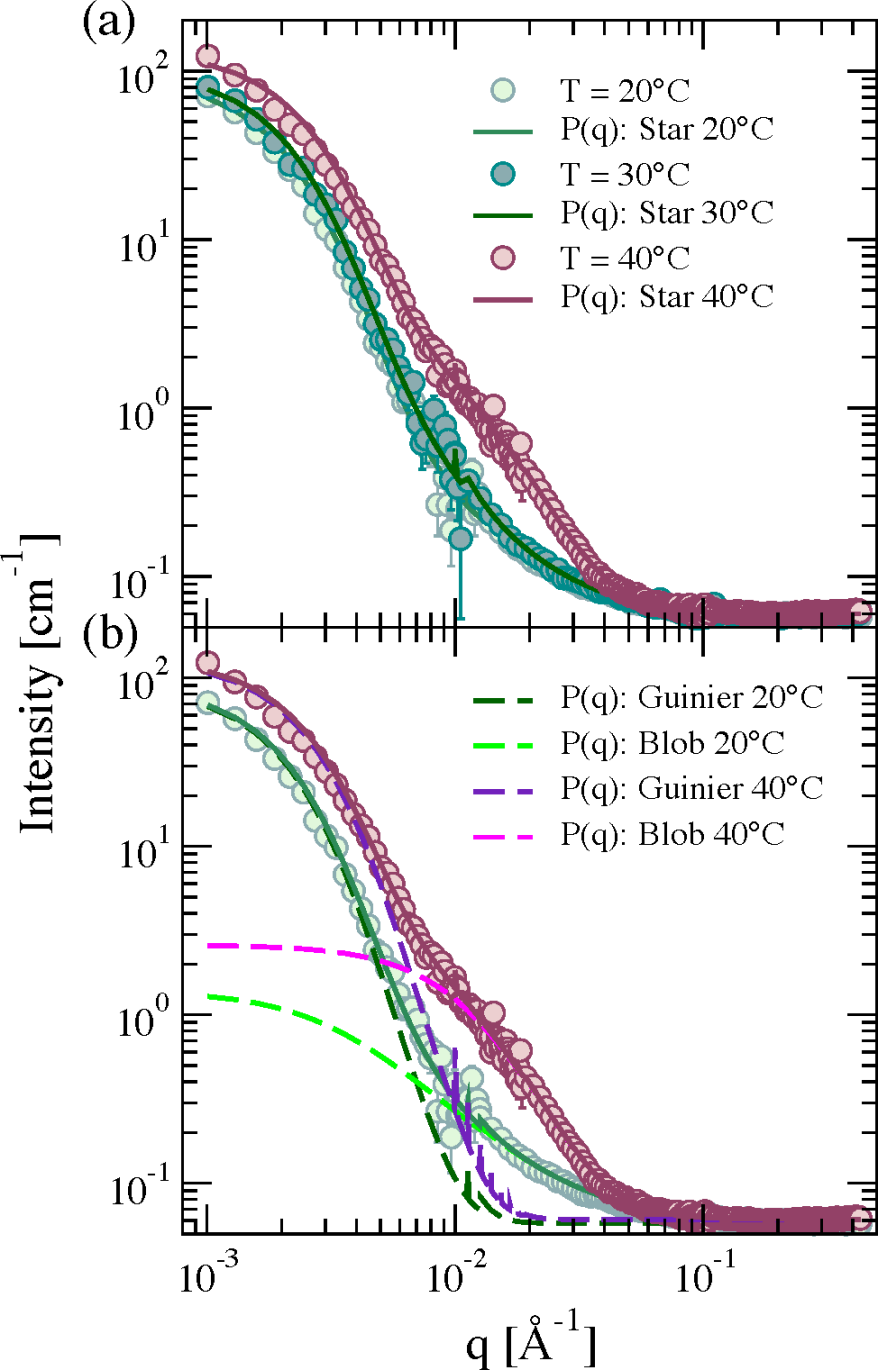
Intensity profiles and fits of sample with *C* = 0.0010 g/mL using the star polymer form factor model: (a) Intensity profiles and fits at 20, 30, and 40 °C; (b) Intensity profiles and fits at 20 and 30 °C and breakdown of the star polymer model in its Guinier and blob scattering components, according to eq 1.

The intensity profiles measured at 20, 30, and 40 °C are reported in Figure 1. Intensities for 20 and 30 °C show a similar shape and the same order of magnitude. They present a smooth decrease as a function of *q* typical of the form factor of diffuse soft structures, like star polymers and loosely cross-linked microgel particles.^33^ Some larger fluctuations observed for 20 and 30 °C at *q* values around 0.01 Å^–1^ are due to a nonperfect overlap between different experimental configurations of the sample–detector distance used to cover the reported range of *q* values. At 40 °C, that is, above the VPT temperature (*T*_c_ ≈ 36 °C), the profile shows a higher intensity and moves to larger *q* values, indicating a reduction in the size of the dispersed particles. Moreover, an additional inflection point at intermediate *q* values (∼2 × 10^–2^ Å^–1^) is present. The results of fitting the experimental SANS intensity profiles using the star polymer model of eq 1 are shown as solid lines in Figure 1a. Note that the fuzzy sphere model^3^ typically used for microgels failed to properly describe the data (Figure S3 of the Supporting Information), in agreement with a recent work where modifications on the topology of a PNIPAM microgel by the presence of an interpenetrating polymer network were also not well-described by the fuzzy sphere model.^46^ At 20 and 30 °C the star polymer model nicely fits the experimental data at all *q* values. There is also good agreement between the model and the measurements at 40 °C but with a slight overestimation of the model for the lowest *q* values. The fitted parameters are listed in Table 2. At 20 and 30 °C the radius of gyration is comparable, *R*_g_ ≈ 900 Å, whereas the blob size reduces from ξ ≈ 350 Å to ξ ≈ 250 Å. The low value of the ratio *R*_g_/*R*_H_ ≈ 0.56 confirms the very open structure of our particles. At 40 °C, *R*_g_ ≈ 740 Å, and the blob size reduces, ξ ≈ 70 Å. The latter is mainly responsible for the appearance of the inflection at intermediate *q* values, as shown in Figure 1b, where the contributions of the two terms in eq 1 are shown separately for 20 and 40 °C. The observed reduction of the radius of gyration and the blob size are in agreement with the expected worsening of the solvent quality, which is confirmed by the increase of μ (Table 2). Note that at 40 °C the ratio *R*_g_/*R*_H_ ≈ 0.63 is consistent with a slightly more compact particle structure.

**Table 2:** Star Polymer Model Parameters Obtained by Fitting the Experimental Intensity Profiles in Figure 1a Using Eq 1^a^

*C* = 0.0010 g/mL			
	20 °C	30 °C	40 °C
A1 (1/cm)	90 ± 10.0	100 ± 7.5	130 ± 10.0
A2 (1/cm)	2.0 ± 0.150	2.0 ± 0.150	1.3 ± 0.125
ξ (Å)	320 ± 10.0	250 ± 12.5	70 ± 3.0
μ	0.66 ± 0.10	1.0 ± 0.05	1.95 ± 0.07
*R*_g_ (Å)	900 ± 20.0	880 ± 15.0	740 ± 22.5
PD	0.25 ± 0.0075	0.25 ± 0.0075	0.25 ± 0.0150

a PD represents the polydispersity index.

SAXS measurements were used to characterize in more detail the *T*-dependent evolution of the particle form factor in the region of intermediate *q* values. The data, which span a *T* range from 25 to 60 °C at intervals of 5 °C, are reported in Figure S1 of the Supporting Information and are thoroughly discussed there. They show that the deswelling transition is progressive and that the inflection observed for the SANS data at 40 °C progressively builds up with increasing *T*, becoming particularly pronounced for *T* > 40 °C.

Next, we use simulations to assess the contribution of the PEG chains to the observed deswelling and blob shrinking.

### Simulations

The experimental form factors obtained by SANS and SAXS indicate a diffuse density profile, compatible with that of a star polymer, and a deswelling behavior that is characterized by a pronounced reduction of the blob size at higher temperatures, which together with the overall particle shrinking leads to the occurrence of an inflection of the form factor at intermediate *q* values. However, because of the small contrast between PNIPAM and PEG, the analysis of the experimental data does not provide a clear indication of the distribution of PEG in the particles and how this affects the deswelling transition. To gain insight on these aspects, we mimicked the experimental system in simulations resolved at the monomer scale.

#### Form Factors: Effect of PEG Distribution

**Figure 2:**
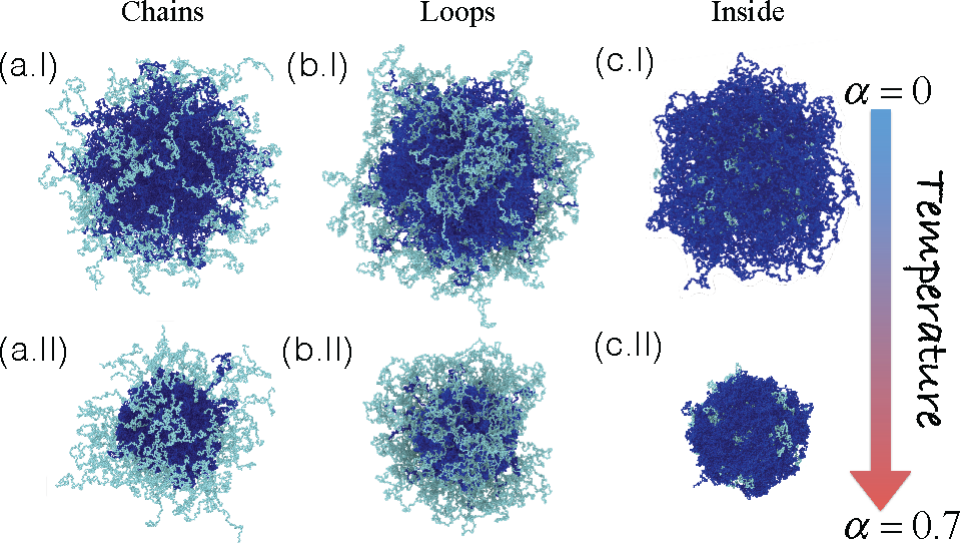
Snapshots illustrating the structural change that the composite microgels with *N*_microgel_ = 42000 beads undergo by increasing temperature when PEG chains are distributed as (a) chains, (b) loops, and (c) inside. Dark blue beads represent the PNIPAM microgel, whereas light blue monomers indicate the PEG polymer chains.

We first study the effect of the PEG distribution on the form factors, looking for the one that leads to results qualitatively comparable to the experimental ones. As detailed in the section Addition of PEG Chains, three different PEG configurations, called chains (PEG linear chains attached at one end to the surface of the PNIPAM particle), loops (PEG linear chains attached at both ends to the PNIPAM particle), and inside (PEG chains inserted inside of the PNIPAM network) are considered. Figure 2 shows snapshots of the resulting composite microgels as a function of the solvophobic parameter α, which is equivalent to the temperature in the experiments. As expected, the composite microgel shows an increasingly more compact core with increasing α because of the thermosensitive nature of the PNIPAM microgel. However, the overall structure is different depending on the PEG distribution. Indeed, in the cases where the PEG chains are protruding from the surface (chains and loops), we observe how an external layer is formed, resembling a core–shell particle. In contrast, in the case where PEG is placed inside the PNIPAM network (inside), no clear difference with a pure PNIPAM microgel can be discerned, except for a few PEG monomers that form sort of patches on the surface, which become more and more evident with increasing temperature (via the effective parameter α).

**Figure 3:**
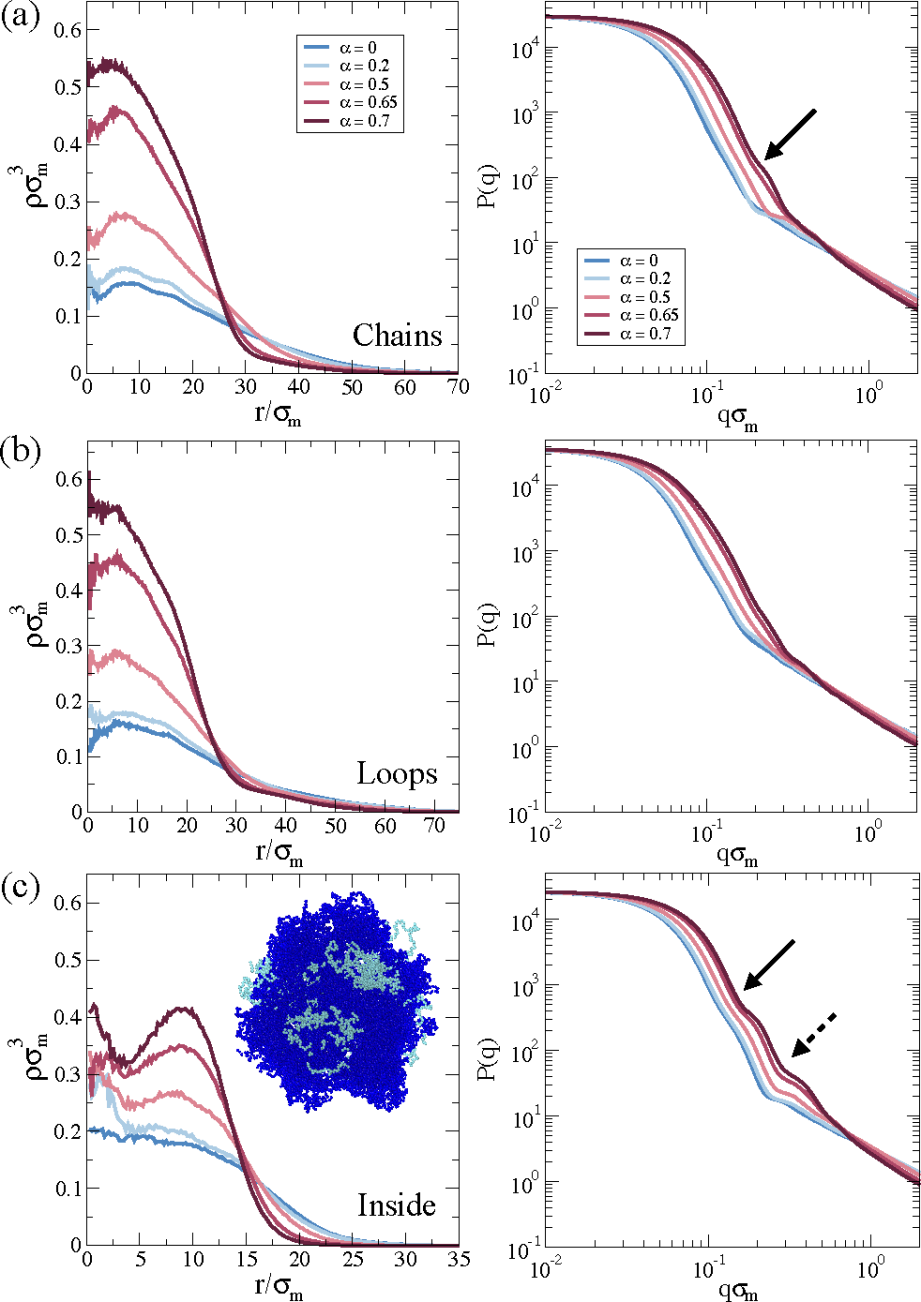
Simulation results for density profiles ρ(*r*) (left column) and form factors *P*(*q*) (right column), averaged over four different composite microgels to roughly mimic the experimental polydispersity, as a function of the solvophobic parameter α for (a) chains, (b) loops, and (c) inside configurations. Solid and dashed arrows in (c, right) highlight the two inflection points discussed in the text. The snapshot in (c) represents a slice of the microgel at α = 0.7 with *N*_microgel_ = 42000 beads.

To gain insight into the structural changes, we calculate the numerical density profiles ρ as a function of α for the three PEG distributions considered. This information is reported in Figure 3. For chains and loops cases, shown in Figure 3a,b, respectively, a dense core is localized at *r* ∼ 10σ_m_ by increasing α. Likewise, in the range 10σ_m_ < *r* ≲ 30σ_m_, the typical corona observed in PNIPAM microgels is appreciated,^37^ followed by a smooth decay corresponding to that of the PEG polymer. Instead, for the inside case, we note that the core develops two peaks with increasing α, indicating that the collapse of the composite microgel is not homogeneous (see Figure 3c and its corresponding inset). The presence of the second peak is attributed to the fluctuations of the PEG chains inside the microgel pushing the PNIPAM outward and, hence, creating a less dense intermediate region. Furthermore, we notice how ρ → 0 at smaller *r* compared to that of chains and loops. In addition to the presence of PEG on the surface, fluctuations of these monomers pull the corona, increasing in this way the total microgel size.

Corresponding form factors *P*(*q*) are shown in the right column of Figure 3. In agreement with the density profiles, the most remarkable structural difference generated by the PEG chains is observed when they are distributed inside the microgel. This result is confirmed by the existence of two inflections in the *P*(*q*) of Figure 3c for the largest α value, analogous to the two peaks observed in the respective ρ(*r*), indicating the presence of two dense regions. The inflections become increasingly pronounced with increasing solvophobicity, in qualitative agreement with the experimental results with increasing *T*. A smaller inflection develops with increasing solvophobicity also when the chains are instead distributed on the surface of PNIPAM and attached at one end (chains). We also explored the situation when PEG chains distributed inside the PNIPAM microgel are attached at only one end of the network, which should more closely resemble the experimental situation. We report this scenario in Figure S4 for a composite PNIPAM microgel with *N*_microgel_ = 42000 beads, comparing the resulting density profiles and form factors, at different effective temperatures, to the case where both ends are connected. In particular, we note that the form factors are almost identical in the two cases, thus allowing us to exclude a crucial influence of either a single or a double end connection of the chains to the network on the present findings.

To link these results to the experimental findings, we notice that the first inflection (present for the chains, loops, and inside cases) arises at *q*σ_m_ ∼ 0.2, while the second one (only present in the inside case) for *q*σ_m_ ∼ 0.4. To compare these numbers with experimental units, we need an estimate for σ_m_, which is the monomer diameter in the simulation. Using the calculated value of *R*_g_ for the *N*_microgel_ = 42000 inside case and setting this equal to the experimental radius of gyration, about 90 nm, we get a rough estimate of σ_m_ for this microgel size of about 2.5 nm. Hence, the two inflections should be located around 8 × 10^–3^ and 1.6 × 10^–2^ Å^–1^, respectively. Although the first one is not present in the experimental data, probably because of the large polydispersity of the microgels, the second one is evident in both SAXS and SANS data. This suggests that the simulated microgel with inside chains is the closest topology to the experimental system.

#### Swelling Behavior

**Figure 4:**
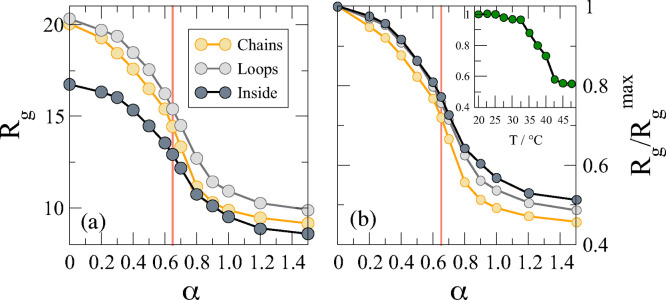
Swelling curves. (a) Radius of gyration *R*_g_ as a function of the solvophobic parameter α for microgels with *N*_microgel_ = 5000 for the three different distributions of PEG polymer chains analyzed in this work; (b) radius of gyration normalized by *R*_g_^max^ to highlight the evolution of particle size as a function of how the chains are distributed. The vertical red line is placed at α ∼ 0.65, indicating the occurrence of the VPT for a pure PNIPAM microgel. Inset: Experimental swelling curve reporting *R*_H_/*R*_H_^max^ versus *T*, where *R*_H_ data are taken from ref (30).

In this section we focus on different swelling stages of the composite microgels upon increasing the solvophobicity parameter for the three different distributions of the PEG chains considered. In Figure 4a, we report the swelling curves of a microgel with *N* = 5000 beads. In agreement with the density profiles discussed in Figure 3, we observe that the composite microgel size is larger when the chains are on the polymer network’s surface. In particular, for the case where both ends are connected to form loops, the composite microgel always acquires a slightly larger size than the case of chains. This situation, which is enhanced by increasing α, resembles what is already observed in charged copolymerized microgels,^18^ where charges tend to swell the network. On the other hand, when the distribution of chains is in the interior, we see that the composite microgel size is the smallest. This is due to both the short chains used in the simulations (see Table 1) and the fact that in this configuration the microgel remains overall more compact.

At the same time, we monitor the volume phase transition (VPT), which has been documented to happen at α ∼ 0.65 for a pure PNIPAM microgel.^37^ Thus, in Figure 4b we normalize the radius of gyration by its value at α = 0, that is, the maximally swollen size, *R*_g_^max^. We can appreciate that the VPT transition seems to occur at slightly larger values of α compared to that of a pure PNIPAM microgel. In particular, particles with polymer chains forming loops on the surface of the microgel and with polymer chains distributed inside show a greater deviation from the VPT observed for pure PNIPAM microgels. Although fluctuations of polymer chains on the surface, as in the loop case, seem to justify this behavior, the shift in the inside case can be attributed to the presence of two dense regions and a depleted region in the composite microgel (Figure 3). The presence of a depleted region prevents PNIPAM monomers from concentrating in the center of the microgel, thus diverting the collapsed regime volume, as we can observe at α = 1.5. The experimental values of *R*_H_/*R*_H_^max^ versus *T*, with *R*_H_^max^ being the hydrodynamic radius measured at *T* = 20 °C, are reported for comparison in the inset of Figure 4b (*R*_H_ data taken from ref (30)). The extent of the *R*_H_ reduction is closely comparable to that found for the inside case in the simulations.

#### Effective Interactions

**Figure 5:**
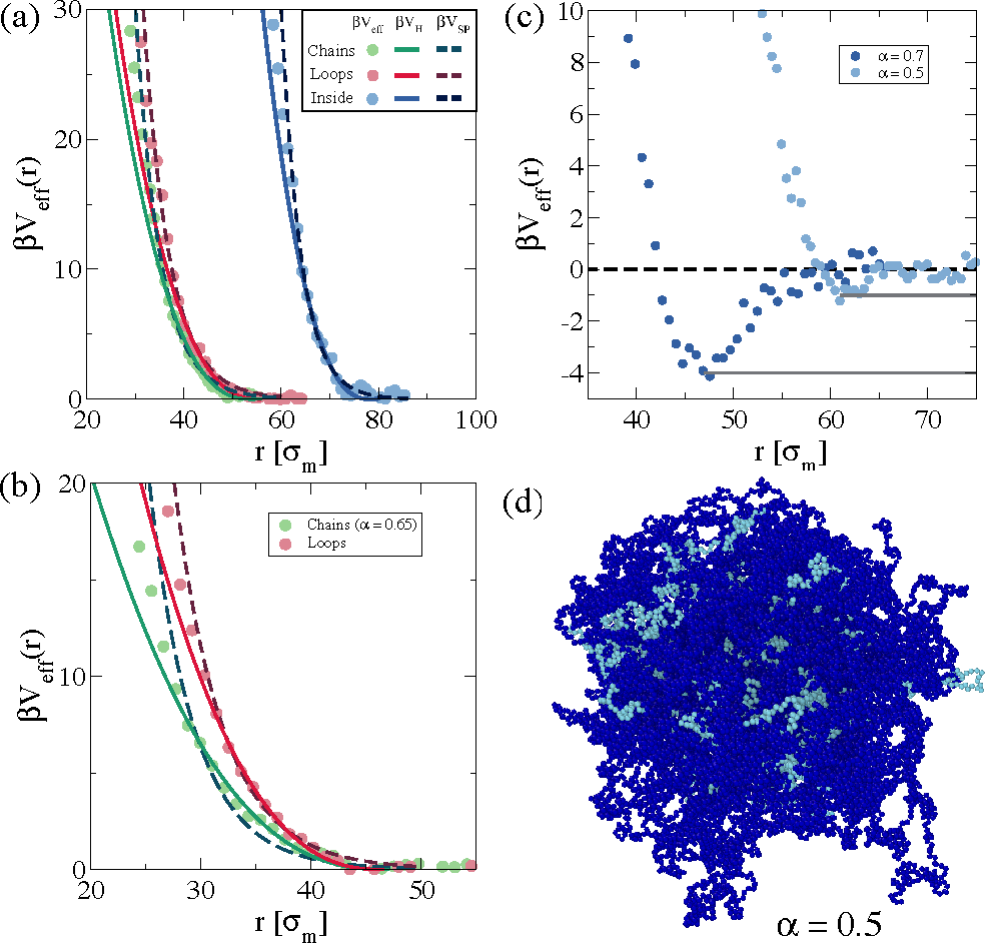
Effective potential β*V*_eff_ calculated through the umbrella sampling technique and fitted by the Hertzian model (eq 10) and the star polymer interaction potential (eq 11) for (a) α = 0 for the three different distributions of the polymer PEG chains, (b) α = 0.65 for the chains and loops cases, and (c) α = 0.5 and α = 0.7 for the inside case. (d) Snapshot of the composite microgel with *N*_microgel_ = 20000 and PEG chains inside at α = 0.5, showing a moderately nonspherical shape because of the fluctuations of the PEG chains inside the PNIPAM microgel. Dark blue beads represent the PNIPAM network, whereas light blue monomers indicate the PEG chains.

We study the effects of PEG chain distribution on the interaction of two PNIPAM–PEG microgels by computing the effective potential β*V*_eff_(*r*). Results are shown in Figure 5 for the three examined cases. The numerical results for the effective interaction at α = 0 are also compared to the expected theoretical expressions. In particular, we consider the Hertzian model, usually employed for microgels,^47−49^ which reads as
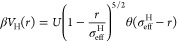
10where *U* is the Hertzian strength related to the elastic energy cost of particle deformation when they are pushed together and σ_eff_^H^ is an effective particle diameter beyond which the interaction vanishes, as indicated by the Heaviside step function θ. Since we earlier noticed that the experimental SANS intensity profiles cannot be fitted by the standard fuzzy sphere model, and thus we had to resort to a star polymer model, we also test whether a star polymer effective potential is applicable to describe the interactions of the particles under investigation. For this reason, we also compare the numerical *V*_eff_ to the star polymer potential developed by Likos and co-workers,^50^ defined as
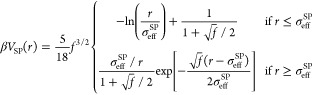
11where *f* is the number of arms, called functionality, and σ_eff_^SP^ represents the characteristic length of a star polymer.

Figure 5a shows β*V*_eff_(*r*) at α = 0 and the fits corresponding to the Hertzian and star polymer potentials. We find that the interaction is repulsive for all PEG distributions, as expected. In particular, for the chains and the loop cases, the star polymer potential is found to properly capture the numerical results in the whole investigated range of *k*_B_*T* using the nominal arm number, *f* = 90, used in the simulations and leaving σ_eff_^SP^ as the only fit parameter. We estimate σ_eff_^SP^ to be ∼28.5σ_m_ and ∼30.0σ_m_ for the chains and loops configurations, respectively. On the other hand, when we fit the data with the Hertzian model, we leave both σ_eff_^H^ and the interactions strength *U* as fit parameters and find that deviations already appear for β*V*_eff_ ≳ 10, analogous to previous observations for standard microgels.^48^ In this case, the estimated interaction lengths are σ_eff_^H^ ∼ 54.0 σ_m_ and ∼52.7σ_m_ for chains and loops, respectively. The remarkable difference in the size of the composite microgel between the two models is expected^51^ since σ_eff_^SP^ is usually well within the region of the outer blob chains, whereas σ_eff_^H^ is approximately twice the hydrodynamic radius *R*_H_.^47^

We now turn our attention to the case in which the PEG chains are distributed within the PNIPAM microgel, the inside case. From Figure 5a we note that the Hertzian model again starts to fail for β*V*_eff_ ∼ 10, with σ_eff_^(H)^ = 78.48σ_m_. On the other hand, the star polymer potential describes the interaction up to β*V*_eff_ ∼ 20 when leaving both *f* and σ_eff_^SP^ as fit parameters, which we find to be *f* = 550 and σ_eff_^SP^ = 51.1σ_m_. Such a large value of the functionality may reflect the difference in the internal density of the particles evidenced in Figure 3c, that is, a heterogeneous internal structure that can be described as a high-functionality star polymer in which the functionality arises from a mixture of the PNIPAM and PEG chains. This is markedly different from previous results on PNIPAM microgels, which even in the case of a low degree of cross-linking could be described with a fuzzy sphere model.

Next, we increase α to study the effective potential close to the VPT. Results for chains and loops are shown in Figure 5b. Previous computational studies of PNIPAM microgels have explored the two-body effective potential up to α = 0.5, that is, below the VPT, to avoid the overall attraction between the two microgels.^48^ However, PEG monomers distributed on the surface turn out to effectively shield this attraction, inducing a completely repulsive interaction even above the VPT temperature, resembling results obtained for amphiphilic microgels.^52^ We thus repeat at high temperatures the analysis made for α = 0 and compare the effective potentials obtained from simulations to the Hertzian and star effective potentials. For chains, we find that the star polymer model (with *f* = 90) is not particularly accurate in the description of the effective potential: this is mainly visible at intermediate distances where *V*_SP_ appreciably underestimates *V*_eff_. Instead, the Hertzian model seems to capture better the interaction in this range, but as usual, only up to ∼10*k*_B_*T*. On the other hand, β*V*_eff_ for loops seems to be well-described by both models. The situation for the inside case at high temperatures is instead completely different, as reported in Figure 5c. Indeed, we find that for α = 0.5 an attraction is already present, with a minimum approximately equal to −1*k*_B_*T*, suggesting the existence of microgel–microgel attractive interactions, even well below the VPT for this system. When we increase the temperature further (α = 0.7), the attraction becomes stronger, reaching approximately −4*k*_B_*T*, as shown in Figure 5c. We highlight that the umbrella sampling technique is not very efficient for studying systems with too strong attractive interactions. Indeed, this technique is based on quantifying the transition probability between different states. Thus, the presence of strong bonds hinders an efficient exploration of the phase space, introducing a bias on the probability transitions. For this reason results at α = 0.7 should be taken with caution. However, for α = 0.5 the involved energies are still not too large and data are reliable.

A microscopic explanation of the onset of the attraction already below the VPT could be related to the fact that in this configuration the increase of *T* induces a higher monomer density close to the external surface of the microgel, resulting in a stronger effect of the solvophobic attraction when two particles are sufficiently close. This idea is supported by the snapshot shown in Figure 5d, where one can see that long PNIPAM chains are present on the surface. In addition, the calculation of the shape anisotropy parameter κ^2^, reported in the Supporting Information (Table S2), suggests that indeed the composite microgel displays a higher degree of anisotropy at intermediate values of α.

Finally, we studied how the presence of PEG affects the elastic properties of the microgels using a method recently proposed by some of us,^48,53^ which is described in the Supporting Information. The resulting moduli are reported in Table S3 and Table S4 in the Supporting Information for α = 0 and α ∼ 0.7, respectively. In particular, we find that the microgels with inside chains are less stiff than the other two cases at α = 0. Also, by increasing α, we observe that all moduli increase. However, results for ν are found to be very noisy, probably due to the fact that they are the results from an indirect estimate through *K* and *G*, and close to ν = 0 at all temperatures. Using the obtained values of the elastic constants, we can perform a more stringent test of the Hertzian model^48,53^ since the Hertzian strength *U* in eq 10 is related to the elastic moduli as . In this way, we can use the estimated values of *Y* and ν, leaving σ_eff_^H^ as the only fit parameter. The fits resulting from this analysis are reported in the Supporting Information (Figure S5), confirming that the Hertzian fit works up to a few *k*_B_*T* for chains and loops, where the extracted σ_eff_^H^ is consistent with the expectations. On the other hand, the fit is very poor for the inside case, where σ_eff_^H^ becomes much larger (contrary to the results, for example, of Figure 4), again supporting the fact that the description with a star polymer potential works better in this case because of the high heterogeneity of the internal structure of a composite microgel with PEG chains inside.

## Summary and Conclusions

Combining scattering experiments and simulations, we investigated the morphology and interactions of composite PNIPAM–PEG particles across the VPT transition. Experimental form factors *P*(*q*) obtained from SANS and SAXS showed a collapse of the composite microgel by increasing *T* beyond *T*_c_. This behavior, also observed for PNIPAM microgels, is accompanied by unusual structural features. In particular, the fuzzy sphere model, which typically provides a good description of the form factor of PNIPAM microgels, fails to describe the PNIPAM–PEG composites. Instead, a star polymer model is able to capture the shape of *P*(*q*). The observed starlike density profile can be the result of the lower degree of cross-linking with respect to PNIPAM microgels investigated in previous studies,^3,54^ leading to a particularly extended and diffuse corona. In addition, the deswelling associated with the increase of *T* is associated with the appearance of an inflection of the experimental form factor at *q* ∼ 10^–2^ Å, which is not observed for simple PNIPAM microgels.

We used simulations to clarify how these unusual structural features are related to the conformations and relative spatial distribution of the PNIPAM and PEG chains. Simulations showed that when the PEG chains are distributed on the external surface of the PNIPAM network, either linked to the surface at one (chains case) or both (loops case) ends, the density profile is characterized by a denser core and a diffuse corona, with the corona progressively shrinking with increasing *T*. When the chains are linked only at one extreme, the decay of the density profile beyond the core becomes particularly sharp for high *T*, leading to the formation of a small inflection in the form factor *P*(*q*) at intermediate *q* values (*q*σ_m_ ∼ 0.1). When the PEG chains are instead located inside the PNIPAM network (inside), the structural evolution is significantly different: the density profile develops two denser regions with increasing *T*, with two separate decays that result in two inflections in *P*(*q*). The *q* value to the second inflection roughly corresponds to that of the experimental form factor. This suggests that the inside configuration is the one more closely resembling the morphology of the experimental system. We should consider however that the latter might present different coexisting configurations, and thus correspond to a combination of the chains and the inside configurations. Indeed, the synthetic method used in this work resembles a precipitation polymerization because at the beginning NIPAM monomers are water-soluble and also PEG-methacrylate chains, so the initial distribution of NIPAM and PEG is random; however, when the PNIPAM chains start to grow, the high polymerization temperature in water results in the tendency of PNIPAM chains to precipitate; however, this is prevented by the PEG-methacrylate stabilization, leading to the progressive building of the core. Further polymerization occurs in a core–shell manner, resulting in a soft core including some PEG chains inside and additional, newly formed PEG chains attached to the surface of the microgel. The fact that the inside configuration significantly differs from common PNIPAM internal structures is also confirmed by the investigation of the elastic properties of microgels, which evidence lower moduli (bulk, shear, and Young modulus) compatible with a less dense/compact particle.

The simulations additionally show a very good agreement with experimental data^30^ concerning the reduction of the particle radius with increasing *T*(α), which is about 50% in both cases. Note that this reduction would be significantly larger in pure PNIPAM particles synthesized with the same method and cross-linking density.^28^ The experimental data in ref (30) correspond to measurements of the hydrodynamic radius using dynamic light scattering. The value of *R*_g_ obtained through the modeling of the SANS experimental data presented here shows a smaller relative reduction, suggesting that the *R*_g_ value estimates the size of the denser core of the particles. Finally, simulations confirm experimental results,^28^ indicating a higher value of *T*_c_ for the PNIPAM–PEG particles.

The presence of PEG chains and their organization also alter the interaction potential between two composite microgels. On one hand, at low *T*, the microgel stiffness is larger in the presence of PEG chains inside the microgel: this is the result of the presence of a softer PEG shell in the chains and loops cases. On the other hand, when *T* increases, the same PEG shell inhibits the attraction induced by the solvophobic character of the PNIPAM polymer network. Instead, for the inside case, the development of such an attraction occurs even below the VPT. This is attributed to the change in the particle density profile observed for this case, which results in a higher density of PNIPAM chains close to the surface. These results indicate that the PEG/PNIPAM composition could be varied to trigger the onset of attractive interactions at well-defined temperatures, even in swollen-like conditions, which is a fascinating possibility in the soft particle realm, very promising for observing new phase and rheological behavior.^2^

## Supporting Information Available

SAXS results, SANS and SAXS intensity profiles comparison, fuzzy sphere model form factor fit, and simulation details of the Hertz modulus (PDF)
