# Supplementary material for: Link between Morphology, Structure, and Interactions of Composite Microgels
Source: Macromolecules. 2022 Feb 14;55(5):1834–43. doi: 10.1021/acs.macromol.1c02171 (PMC8908736; doi:10.1021/acs.macromol.1c02171)
Supplement: Supplementary file 1 — ma1c02171_si_001.pdf [file ma1c02171_si_001.pdf]

# Supplementary material : Link between morphology, structure and interactions of composite microgels


Rodrigo Rivas-Barbosa[a,b,†], José Ruiz-Franco[a,c,d†], Mayra A. Lara-Peña[b], Jacopo Cardellini[e], Angel Licea-Claverie[f], Fabrizio Camerin[c,a], Emanuela Zaccarelli* [c,a], and Marco Laurati* [e]

[a]Department of Physics, Sapienza University of Rome, Piazzale Aldo Moro 2, 00185 Roma, Italy
[b]División de Ciencias e Ingenierías, Universidad de Guanajuato, Lomas del Bosque 103, 37150 León, Mexico
[c]CNR Institute of Complex Systems, Uos Sapienza, Piazzale Aldo Moro 2, 00185, Roma, Italy
[d]Physical Chemistry and Soft Matter, Wageningen University & Research, Stippeneng 4, 6708WE Wageningen, The Netherlands
[e]Dipartimento di Chimica and CSGI, Università di Firenze, 50019 Sesto Fiorentino, Italy
[f]Centro de Graduados e Investigación en Química del Tecnológico Nacional de México/Instituto Tecnológico de Tijuana, 22500 Tijuana, Mexico


## SI. SAXS: DETAILED DESWELLING BEHAVIOR

Additional SAXS measurements of the intermediate $q$ range were performed to follow more in detail the deswelling behavior of the microgel particles. The intensity profiles obtained at 8 different temperatures are presented in Fig. S1. At low $q$ values , near 0.02 $\text{Å}^{-1}$, profiles can be separated in three groups according to the intensity in this region: *(i)* the first group includes profiles for 25 °C, 30 °C and 35 °C, which are very similar; *(ii)* the second group is formed by the profiles corresponding to 40 °C and 45 °C, which present a significantly larger scattering which increases with $T$, and the inflection already observed in the SANS data; finally *(iii)* the third group is formed by profiles corresponding to 50 °C, 55 °C and 60 °C, which present the largest scattering and the most pronounced inflection, but are now comparable with each other. Remembering that the critical temperature for these particles is $T_c \approx 36$ °C, we may speculate that samples in the SAXS measurements were not fully equilibrated at the nominal temperature and that the real temperature of the measurements might be nearly 5 °C smaller than the nominal value. This assumption would be consistent with the idea that temperatures of group *(i)* lie below, temperatures of group *(ii)* around, and temperatures of group *(iii)* above the VPT temperature. This is supported by the comparison of SAXS and SANS data presented in section SII, which shows that SAXS data for 25 °C, 35 °C and 45 °C overlap with SANS data for 20 °C, 30 °C and 40 °C, respectively. The SAXS data complement the SANS data showing that the deswelling transition is progressive and leads to the inflection of the form factor at intermediate $q$ values.

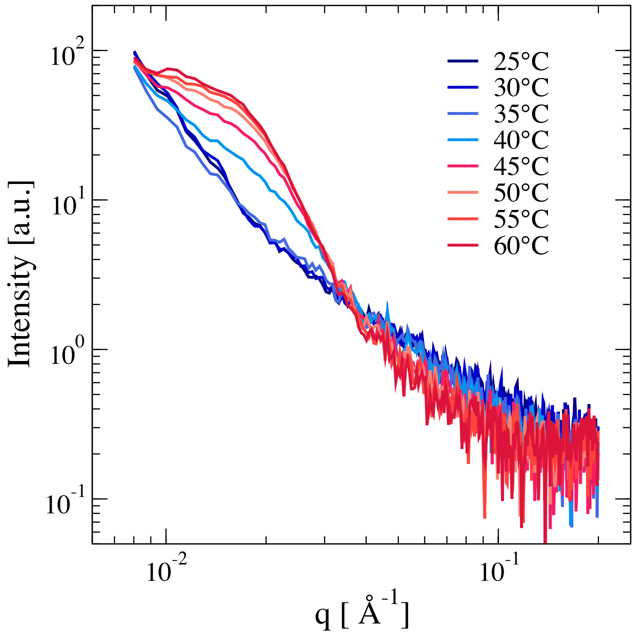


Figure S1. Intensity profiles obtained from SAXS at different temperatures.

## SII. COMPARISON OF SANS AND SAXS RESULTS

Fig. S2 shows a comparison of the SANS and SAXS measurements after the SAXS data sets have been scaled in the *y*-axis plus added the SANS background. After applying scaling and shifting, excellent qualitative agreement between the profiles obtained with the two techniques is observed in the overlapping $q$ range, when SAXS data are compared with SANS data at a temperature about 5 °C lower. This supports the speculation that the effective sample temperature of the SAXS measurements was about 5 °C lower than the nominal temperature.

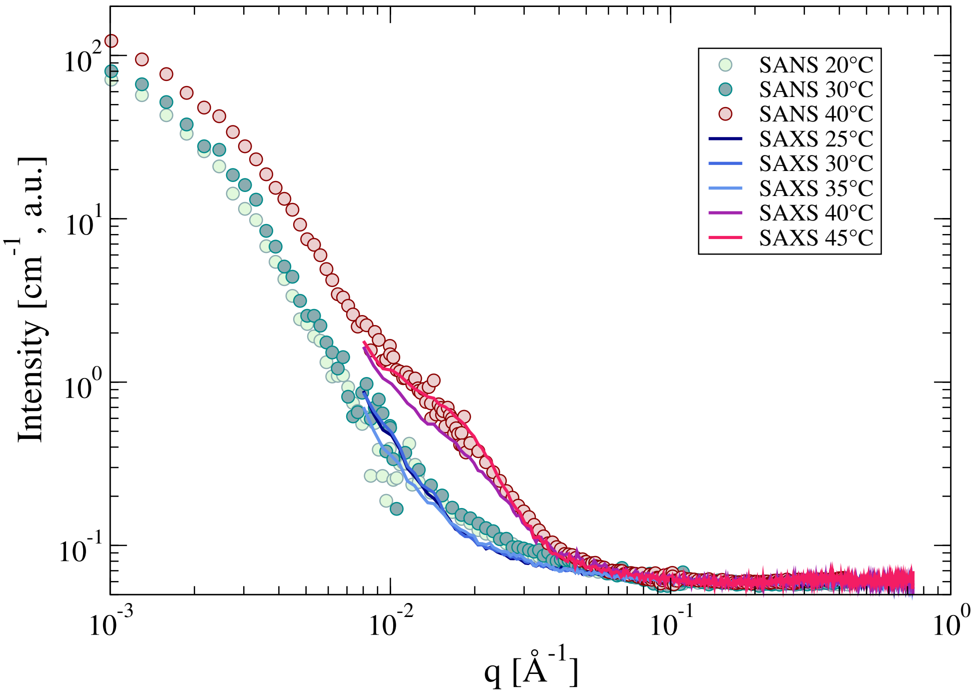


Figure S2. SANS and SAXS intensity profiles. The SAXS data has been scaled with different parameters for the (25°C, 30°C, 35°C) and (40°C, 45°C) sets. SANS intensity in cm$^{-1}$ units, SAXS intensity in arbitrary units.

## SIII. FUZZY SPHERE

Figure S3 and table S1 show the results of fitting with the fuzzy sphere model[1] the SANS intensity profiles. The fuzzy sphere model is given by

$$P(q) = \frac{3\left[\sin(qR) - qR\cos(qR)\right]}{(qR)^3} \exp\left(\frac{-(\sigma q)^2}{2}\right) \tag{S1}$$

The fuzzy sphere model is unable to fit the intensity profiles, specially at the medium to large $q$ values. The unsuccessful results of this model led us to choose the star polymer model as described in the manuscript.

| | 20°C | 30°C | 40°C |
|---|---|---|---|
| $R$ (Å) | 700 | 650 | 650 |
| $\sigma$ (Å) | 100 | 80 | 25 |

Table S1. Parameters of the fuzzy sphere model of Eq. S1 obtained by fitting the experimental intensity profiles.

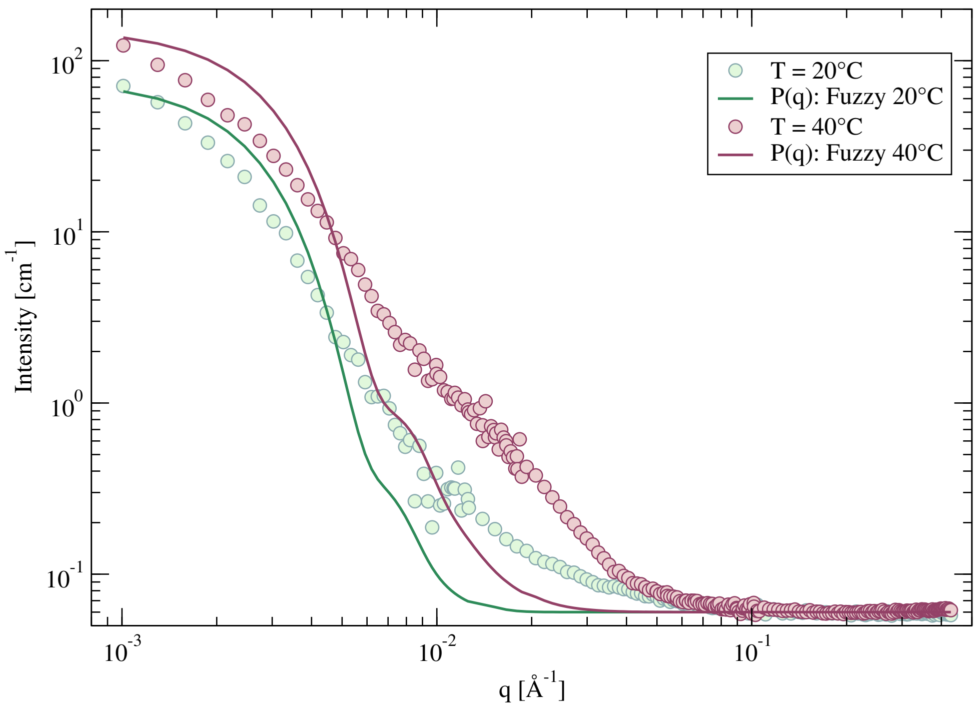


Figure S3. Intensity profiles and fits using the fuzzy sphere model.

## SIV. INSIDE PEG CHAINS DISTRIBUTION ATTACHED AT ONLY ONE END OF THE PNIPAM MICROGEL.

In Fig. S4(a) and Fig. S4(b) we report the density profile $\rho(r)$ and form factor $P(q)$, respectively, for a $N_{microgel} = 42000$ microgel where PEG chains distributed inside of the PNIPAM network are attached either at one or at both ends (the latter being the case reported in the manuscript). While we find some small changes in $\rho(r)$ near the core at high effective temperatures ($\alpha = 0.7$), the single/double connection of the chains does not appreciably affect $P(q)$.

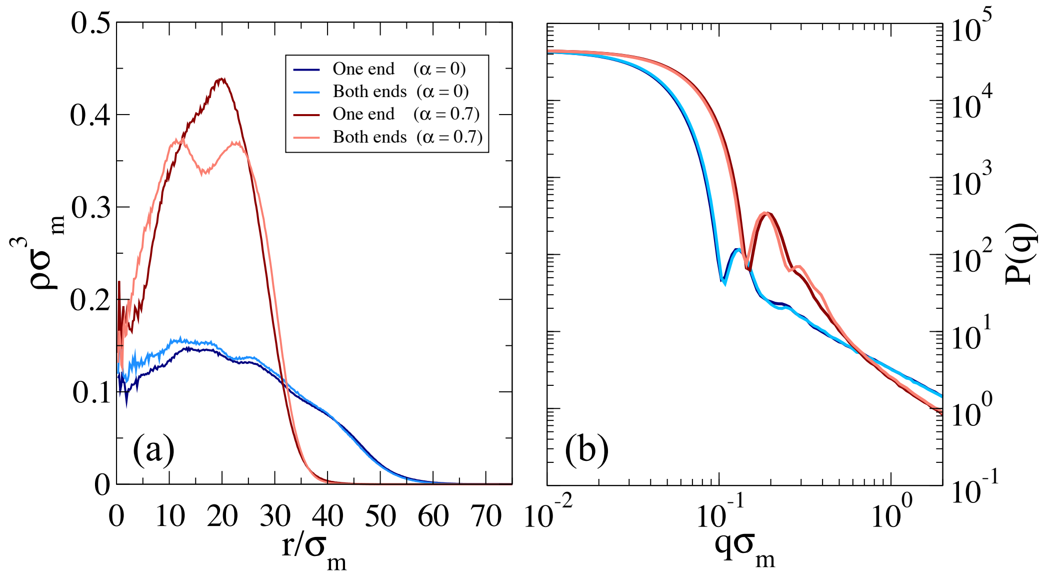


Figure S4. (a) Density profiles $\rho(r)$ and (b) numerical form factors $P(q)$ for a PNIPAM composite microgel with $N_{microgel} = 42000$ beads and PEG chains inside the microgel, considering that chains are attached only with one or both ends to the network, at different $\alpha$ values.

## SV. SHAPE ANISOTROPY

We report the shape anisotropy parameter $\kappa_2$ for a composite PNIPAM microgel with $N_{microgel} = 20000$ beads as a function $\alpha$, for the three PEG chains distributions considered here. This parameter is defined as

$$\kappa^2 = 1 - 3\left\langle \frac{\lambda_1 + \lambda_2 + \lambda_3}{\left(\lambda_1\lambda_2 + \lambda_2\lambda_3 + \lambda_3\lambda_1\right)^2} \right\rangle \tag{S2}$$

where $\lambda_1 \geq \lambda_2 \geq \lambda_3$ are the three eigenvalues from the gyration tensor.[2]

| $\alpha$ | 0 | 0.2 | 0.5 | 0.65 | 0.7 |
|---|---|---|---|---|---|
| $\kappa^2_{chains} \times \left(10^{-3}\right)$ | 5.7 | 4.1 | 6.3 | 3.7 | 19.7 |
| $\kappa^2_{loops} \times \left(10^{-3}\right)$ | 5.5 | 1.6 | 8.6 | 5.3 | 2.9 |
| $\kappa^2_{inside} \times \left(10^{-3}\right)$ | 8.0 | 8.4 | 12.0 | 12.0 | 9.5 |

Table S2. Shape anisotropy parameter $\kappa^2$ for a composite PNIPAM microgel with $N_{microgel} = 20000$ beads as a function of the PEG chains distribution and the effective temperature $\alpha$.

## SVI. CALCULATION OF THE ELASTIC MODULI AND HERTZIAN POTENTIAL FITS

To calculate the microgel elastic moduli, we follow the approach that some of us recently developed.[3,4] From Mooney-Rivlin theory, the energy $U$ for a three-dimensional object due to thermal fluctuations can be written as a function of the invariants $J$ and $I$ of the strain tensor as

$$U\left(J, I_1, I_2\right) = U_0 + W\left(J\right) + W\left(I_1\right) + W\left(I_2\right)\ , \tag{S3}$$

where $U_0$ is the energy of a reference configuration. This configuration is approximated to an average ellipsoid with semi-axes $a_1$, $a_2$ and $a_3$, obtained by the gyration tensor built via the convex hull, defined on a set of points that encompasses all the particles of the composite microgel. These points are used for computing the gyration tensor, and hence, its diagonalization provide the three eigenvalues $\lambda_1$, $\lambda_2$ and $\lambda_3$, sorted from the largest to the smallest. Thus, the semi-axes are defined as $a_i = \sqrt{3\lambda_i}$. Likewise, $W$ depends on the potential of mean force, defined as

$$W\left(X\right) = -k_B T ln P\left(X\right) + D_X \tag{S4}$$

where $X = J, I_1, I_2$, $P\left(X\right)$ represents the respective probability distribution and $D_X$ is an arbitrary constant. These potentials can be fitted by using

$$f\left(X; M_X, X_0, \gamma, C\right) = M_X\left(X - X_0\right)^{\gamma} + C \tag{S5}$$

with $\gamma = 2$ for $X = J$ and $\gamma = 1$ for $X = I$. From $M_J$ and $M_I$ we obtain the bulk modulus $K$ and the shear modulus $G$ as,

$$K = \frac{2M_J}{V} \tag{S6}$$

$$G = \frac{2M_I}{V}. \tag{S7}$$

The knowledge of these two elastic moduli allows us to calculate also the Young modulus $Y$ and the Poisson's ratio $\nu$ as

$$Y = \frac{9KG}{3K + G} \tag{S8}$$

$$\nu = \frac{3K - 2G}{2\left(3K + G\right)}. \tag{S9}$$

The results for the elastic moduli of the simulated microgels are reported in Table S3 for $\alpha = 0$ and in Table S4 for $\alpha \sim 0.7$, respectively. We note that the error for $K$ and $G$, that are directly estimated from the simulations is $\lesssim 10\%$, while this is much larger for $Y$ and, particularly, for $\nu$ that is found to be close to zero, being sensibly smaller, particularly for the inside case, than the typical values found for standard microgels,[3] particularly at large $\alpha$. To have a better estimate of the Poisson's ratio, we would probably need to average over different microgel topologies, but, for the purpose of this paper, we notice that $\nu$ does not contribute much to the fit of the Hertzian strength, and thus we avoid making strong claims on its values.

| $\alpha = 0$ | $K\ (\times 10^{-3})$ | $G\ (\times 10^{-3})$ | Y $(\times 10^{-3})$ | $\nu$ |
|---|---|---|---|---|
| Chains | 1.68 | 1.97 | 4.25 | 0.08 |
| Loops | 1.25 | 1.66 | 3.45 | 0.04 |
| Inside | 0.70 | 1.21 | 2.30 | −0.05 |

Table S3. Elastic moduli for the composite microgel changing the polymer PEG chain distribution in units $k_B T/\sigma_m^3$. Whereas the error for $K$ and $G$ is $\lesssim 10\%$, for $Y$ and $\nu$ it exceeds $\sim 20\%$.

| | $K\ (\times 10^{-3})$ | $G\ (\times 10^{-3})$ | Y $(\times 10^{-3})$ | $\nu$ |
|---|---|---|---|---|
| Chains ($\alpha = 0.65$) | 3.46 | 2.58 | 6.20 | 0.2 |
| Loops ($\alpha = 0.65$) | 2.92 | 6.62 | 11.37 | −0.14 |
| Inside ($\alpha = 0.7$) | 2.84 | 4.32 | 8.72 | 0.01 |

Table S4. Elastic moduli for the composite microgel changing the polymer PEG chain distribution, in units $k_B T/\sigma_m^3$. Whereas the error for $K$ and $G$ is $\lesssim 10\%$, for $Y$ and $\nu$ it exceeds $\sim 20\%$.

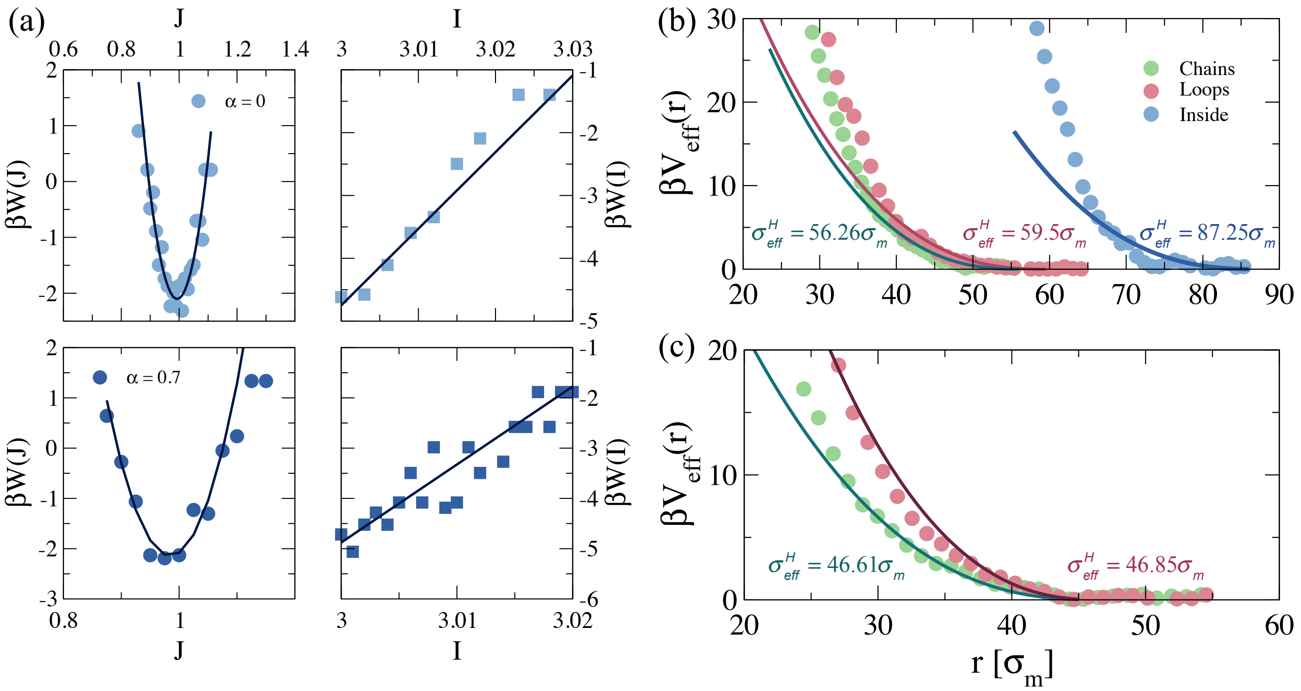


Figure S5. (a) Potential of mean force $W(J)$ and $W(I)$ for a composite microgel with polymer PEG chains inside at $\alpha = 0$ and 0.7. Here, symbols are simulation data, whereas solid lines are fits according to Eq. S5. Effective potential interaction $\beta V_{eff}(r)$, where symbols are simulation results and solid lines are fits with the Hertzian model considering elastic moduli at (b) $\alpha = 0$ and (c) $\alpha = 0.65$

To translate these values into experimental units, we need an estimate of $\sigma_m$ for the simulated microgel. For the $N_{microgel} = 20K$ "inside" microgel, a comparison of the radii of gyration yields $\sigma_m \sim 3$ nm. Instead, such an estimate cannot be obtained for chains and loops microgels, because they shrink differently with respect to the experimental ones and, thus, we cannot meaningfully extract a value of $\sigma_m$ that is independent of $\alpha$. Limiting ourselves to the inside case, we find that the moduli are: $K \sim 107$ Pa, $G \sim 183$ Pa and $Y \sim 350$ Pa for low temperatures ($\alpha = 0$), while they are $K \sim 447$ Pa, $G \sim 680$ Pa and $Y \sim 1372$ Pa close to the VPT

($\alpha = 0.7$, which should correspond to roughly T=35 °C). These values are considerably smaller than what usually estimated in experiments for standard microgels. This can be attributed, on one hand, to the smaller moduli found for the inside case with respect to the other microgels (and if we compare with our previous paper for standard microgels[3] at a comparable $N_{microgel}$) as well as to the low crosslinker concentration used in this work (c=1%). In addition, we notice that our estimates, being based on fluctuations, mostly capture the moduli of the corona, which is expected to be smaller than that of the whole microgel.

Finally, Fig. S5(a) shows the potential mean force $W(J)$ and $W(I)$ for a composite microgel with PEG chains distributed inside of the PNIPAM microgel at $\alpha = 0$ and $\alpha = 0.7$, with the respective fits (Eq. S5). In Fig. S5(b) and (c), we show the fit of the effective potential $\beta V_{eff}$, computed by umbrella sampling, with the Hertzian model defined as

$$\beta V_H(r) = \frac{2Y {\sigma_{eff}^H}^3}{15(1-\nu^2)} \left(1 - \frac{r}{\sigma_{eff}^H}\right)^{5/2} \theta\left(\sigma_{eff}^H - r\right), \tag{S10}$$

Here, the effective Hertzian particle diameter $\sigma_{eff}^H$ is a fit parameter, while the elastic moduli $Y$ and $\nu$ are fixed to the values calculated by simulations.

## REFERENCES


[1] Stieger, M., Richtering, W., Pedersen, J. S., and Lindner, P., "Small-angle neutron scattering study of structural changes in temperature sensitive microgel colloids," *The Journal of chemical physics* **120**(13), 6197–6206 (2004).

[2] Rovigatti, L., Capone, B., and Likos, C. N., "Soft self-assembled nanoparticles with temperature-dependent properties," *Nanoscale* **8**(6), 3288–3295 (2016).

[3] Rovigatti, L., Gnan, N., Ninarello, A., and Zaccarelli, E., "Connecting elasticity and effective interactions of neutral microgels: The validity of the hertzian model," *Macromolecules* **52**(13), 4895–4906 (2019).

[4] Camerin, F., Gnan, N., Ruiz-Franco, J., Ninarello, A., Rovigatti, L., and Zaccarelli, E., "Microgels at interfaces behave as 2d elastic particles featuring reentrant dynamics," *Physical Review X* **10**(3), 031012 (2020).
